# Polyphenol Bioconversion and Volatile Aroma Production by *Meyerozyma* sp. C3 Isolated from Kombucha in the Theaflavin–Sucrose Fermentation Model

**DOI:** 10.3390/foods15193453

**Published:** 2026-09-27

**Authors:** Tong Tong, Jiayu Wu, Shengyu Jia, Chixin Liao, Hengyu Zhang, Jianlian Xie, Wenxi Chen, Yutong Huang, Xiaotong Wang, Xinna Wu, Yuzhen Zhou, Yongjin He, Kai Zhao

**Affiliations:** 1 Fujian Key Laboratory of Developmental and Neural Biology, College of Life Sciences, Fujian Normal University, Fuzhou 350117, Chinayongjinhe@fjnu.edu.cn (Y.H.); 2 The Strait Floral Industry Science and Technology Innovation Hub, Key Laboratory of National Forestry and Grassland Administration for Orchid Conservation and Utilization, College of Landscape Architecture and Art, Fujian Agriculture and Forestry University, Fuzhou 350002, China

**Keywords:** kombucha, metagenomics, theaflavins, fermentation, microbial community reconstruction, yeast

## Abstract

This study integrated metagenomic and untargeted metabolomics analysis to characterize differences in kombucha communities and metabolites between black tea–sucrose and theaflavin–sucrose systems. Theaflavins selectively enriched yeasts, particularly *Meyerozyma* and *Lodderomyces*, and increased the annotated abundance of carbohydrate-metabolism genes, glycoside hydrolases, and glycosyltransferases. Based on these community-level findings, the newly isolated *Meyerozyma* sp. C3 was employed to establish a simplified single-strain fermentation model to investigate the biotransformation potential of the enriched yeast taxon. The strain exhibited stable growth and sustained acidification throughout fermentation, with a theaflavin conversion rate of 39.5% after 7 days. Metabolomic analysis revealed substantial changes in oligosaccharides (e.g., maltotriose, raffinose), flavonoids/flavanes (e.g., epigallocatechin gallate, (+)-gallocatechin, (-)-epigallocatechin, and catechin derivatives), and organic acids (e.g., citric and isocitric acid), while volatile profiling identified 2-phenylethanol and 4-ethylphenol as newly dominant aroma compounds after fermentation. These results indicate that a yeast-driven route for theaflavin transformation and flavor formation, supporting targeted strain mining and functional fermented-tea development.

## 1. Introduction

Kombucha is a tea-based fermented beverage produced through fermentation of sugared tea by a symbiotic culture of bacteria and yeasts (SCOBY), commonly referred to as tea fungus, which contains yeasts, acetic acid bacteria, lactic acid bacteria, and other microorganisms [1]. During fermentation, this consortium transforms major black-tea constituents and generates a spectrum of dominant metabolites. Kombucha has been reported to exhibit anti-inflammatory and antimicrobial activities [2,3,4]. However, its quality and functionality depend not only on microbial symbiosis but also strongly on the type oftea type, carbon-source composition, and other substrate constituents, all of which can modulate strain growth, community equilibrium, and metabolite profiles [5,6].

Theaflavins are tea-derived beneficial polyphenols that also contribute to the color, aroma, quality, and physiological functionality of black tea [7]. These compounds share a characteristic benzotropolone skeleton and are primarily present as four major constituents [7,8]. These compounds exert regulatory effects in multiple disease contexts and have been reported to show potential preventive or therapeutic activities against cardiovascular disease, diabetes, as well as antioxidant and antimutagenic properties etc. [9,10,11]. However, their structural complexity, high degree of polymerization, and relatively large molecular size may limit their absorption, metabolism, and utilization in human body [12]. The nutritional effects of dietary plant polyphenols are closely associated with their microbial degradation and biotransformation, processes that occur both in the gastrointestinal tract in vivo and in food-fermentation systems [5,13,14,15]. In the intestinal context, theaflavins are poorly absorbed in the small intestine and exhibit limited bioavailability. Consequently, a substantial proportion reaches the colon, where they are transformed by the gut microbiota into lower-molecular-weight phenolic compounds through degalloylation, C-ring cleavage, oxidation, and related reactions, suggesting that microbiota-mediated biotransformation represents an important mechanism underlying the biological activities of theaflavins in vivo [16,17,18,19]. Beyond the intestinal environment, microbial transformation of polyphenols is also an important feature of food fermentation. The metabolic activities of fermenting microorganisms can alter the composition and chemical structures of polyphenols and consequently reshape the metabolite profile of the fermentation system [13,14]. Tea fermentation provides a particularly relevant food-based context for examining this process. Previous studies have shown that pure-culture solid-state fermentation of dark tea with *Eurotium cristatum* leads to a progressive decrease in theaflavin content, accompanied by extensive changes in phenolic and flavonoid metabolites. Similarly, liquid-state fermentation of dark tea with different Aspergillus species results in marked species-dependent differences in theaflavin levels and overall chemical composition [20,21]. In addition, yeasts and other microorganisms associated with kombucha have been reported to contribute to the biotransformation of tea polyphenols [22,23]. Although these studies suggest that theaflavins may undergo microbially mediated transformation during fermentation, the characteristics of theaflavin biotransformation and the key functional strains involved remain poorly understood within the complex symbiotic fermentation system of kombucha.

This study employed a theaflavin–sucrose system as a simplified experimental model rather than as a representation of conventional kombucha fermentation. Theaflavins were added as the target polyphenolic constituents. This simplified design was intended to reduce the interference arising from the multiple polyphenols and other bioactive compounds naturally present in tea, thereby allowing the microbial responses and biotransformation associated with theaflavins to be examined under controlled conditions. Accordingly, two main questions were addressed: (1) whether theaflavins exert selective regulatory effects on the kombucha microbial community and (2) whether the dominant microorganisms enriched in the presence of theaflavins possess the capacity of biotransformation. To address these questions, this study integrated metagenomic and untargeted metabolomic approaches to compare microbial community composition and functional gene profiles between black tea–sucrose and theaflavin–sucrose kombucha systems, enabling the screening of theaflavin-enriched core functional microorganisms and establishing functional links between core strains and key metabolic signatures. The identification of strains capable of efficient theaflavin transformation could enable targeted fermentation strategies for producing premium tea beverages with standardized bioactive profiles and improved health-promoting properties.

## 2. Materials and Methods

### 2.1. Sample Preparation

The original kombucha starter culture and the yeast strain *Meyerozyma* sp. C3 (Supplementary Figure S3) isolated from this starter culture were preserved in the Microbiology Laboratory, College of Life Sciences, Fujian Normal University, China [24]. Before use, *Meyerozyma* sp. C3 was pre-cultured at 28 °C, and the cell density of the resulting suspension was determined using a hemocytometer. The cell suspension was then adjusted to 9–10 × 10^7^ cells/mL with sterile distilled water. Theaflavins were purchased from Guangdong Kangda Biotechnology Co., Ltd. (Guangzhou, China). Yeast extract, peptone, anhydrous glucose, and agar were purchased from Sinopharm Chemical Reagent Co., Ltd. (Shanghai, China). Methanol, formic acid, and acetonitrile were of chromatographic grade.

The yeast medium consisted of yeast extract (10 g/L), peptone (15 g/L), anhydrous glucose (20 g/L), and agar (20 g/L). The black tea–sucrose medium contained black tea (6 g/L) and white granulated sugar (60 g/L). The theaflavin concentration of 0.048 g/L was selected to approximate the theaflavin level corresponding to the black tea concentration used in the comparative fermentation system, based on a theaflavin content of approximately 0.8% in black tea, and white granulated sugar was added at a concentration of 60 g/L [25]. All media were prepared with distilled water and sterilized before use.

### 2.2. Determination of Yeast Growth Dynamics and Physicochemical Properties During Fermentation

The activated *Meyerozyma* sp. C3 suspension was inoculated into the theaflavin fermentation medium at an inoculum size of 1% and incubated under static conditions at 28 °C for 15 days. During fermentation, samples were aseptically withdrawn at 48 h intervals, and yeast growth was assessed by determining the optical density at 600 nm. System pH was determined using a PHS-3C pH meter (Shanghai INESA Scientific Instrument Co., Ltd., Shanghai, China). Total acidity was determined using acid–base indicator titration in accordance with GB 12456-2021, the National Food Safety Standard for the Determination of Total Acid in Foods [26], and the results were calculated as acetic acid equivalents [25].

The theaflavin content was determined using a systematic analytical method [27], involving sequential extraction with ethyl acetate and *n*-butanol, with absorbance measured at 380 nm using 95% ethanol as the blank reference. Each treatment included three biological replicates. An uninoculated medium incubated under the same conditions was used as the unfermented control.

### 2.3. DNA Extraction

Black tea and theaflavins were separately used as fermentation substrates and supplemented with white granulated sugar to prepare liquid fermentation media for kombucha cultivation. An aliquot of 200 mL of the preserved original kombucha starter culture was combined with 800 mL of the prepared liquid fermentation medium. Each vessel was then sealed with sterile gauze and maintained under static conditions at ambient temperature. For each fermentation system (BP and TF), three independent fermentation vessels were prepared as biological replicates. On day 5, equal volumes of fermentation broth from the three replicate vessels were pooled prior to centrifugation and DNA extraction to obtain a representative sample for metagenomic sequencing.

Based on preliminary growth curve analysis [28], the kombucha SCOBY reached peak microbial density at day 5, indicating maximal microbial activity optimal for capturing core community composition responsive to substrate differences. Original kombucha fermentation broths from the black tea–sucrose system (BP) and the theaflavin–sucrose system (TF) were collected on day 5. After centrifugation, the pellet was retained for genomic DNA extraction with the PowerSoil^®^ DNA Isolation Kit(MO BIO Laboratories, Carlsbad, CA, USA). DNA concentration was measured using a Qubit^®^ 2.0 fluorometer (Life Technologies Corporation, Carlsbad, CA, USA), and sequencing libraries were prepared from the purified DNA with the NEBNext^®^ Ultra™ DNA Library Prep Kit for Illumina^®^ (New England Biolabs, Inc., Ipswich, MA, USA).

### 2.4. Metagenomic Sequencing and Bioinformatic Analysis

Whole-genome metagenomic sequencing was conducted on the Illumina platform. Raw reads underwent quality evaluation with FastQC and were processed with Trimmomatic to remove adapters and low-quality sequences. High-quality reads were assembled using IDBA_UD. Open reading frames (ORFs) ≥ 100 bp were predicted using Prodigal, translated into amino acid sequences, and dereplicated using CD-HIT. Clean reads were mapped to the non-redundant gene catalog using Bowtie 2, and mapped reads were quantified using SAMtools. Gene abundance was normalized according to mapped-read counts and gene length, and functional abundance was subsequently calculated based on the abundance of genes assigned to each functional category. The resulting non-redundant protein sequences were aligned against the NR, KEGG, CAZy, and SEED databases using DIAMOND with the blastp algorithm to obtain taxonomic annotations and identify metabolic functional pathways.

### 2.5. LC–MS Analysis

Samples collected on day 7 were chosen for metabolomic profiling [25], representing a mid-fermentation timepoint that balances sufficient microbial transformation with minimal late-stage product degradation [29]. For LC-MS analysis, the fermentation broth from the theaflavin–sucrose system inoculated with *Meyerozyma* sp. C3 was designated as the C3 group, whereas the corresponding uninoculated control was designated as the Blank group. LC-MS analysis was conducted with reference to a previously reported method [30], with modifications to sample preparation, chromatographic separation, and mass spectrometric conditions. Brirfly 1.0 mL of each sample was freeze-dried and reconstituted in 400 μL of pre-chilled methanol/water (4:1, *v*/*v*), with 20 μL of L-2-chlorophenylalanine (0.3 mg/mL in methanol) added as the internal standard. The samples were sonicated for 3 min and centrifuged at 13,000 rpm for 10 min at 4 °C. The supernatant was then filtered through a 0.22 μm membrane prior to LC–MS analysis.

An ACQUITY UPLC HSS T3 column (100 mm × 2.1 mm, 1.8 μm) at 45 °C was used with mobile phases of 0.1% formic acid in water (A) and 0.1% formic acid in acetonitrile (B) at 0.35 mL/min. The gradient program was: 5% B (0.01–2 min), 5–30% B (2–4 min), 30–50% B (4–8 min), 50–80% B (8–10 min), 80–100% B (10–14 min), 100% B (14–15 min), 100–5% B (15–15.1 min), and 5% B (15.1–16 min). Injection volume was 2 μL. MS analysis used ESI in positive and negative modes (m/z 125–1000) with spray voltage +3500/−3000 V, resolutions 70,000 (full-scan) and 17,500 FWHM (HCD MS/MS), capillary temperature 320 °C, sheath gas +40/−35 Arb, and auxiliary gas +10/−8 Arb.

### 2.6. Headspace Solid-Phase Microextraction Coupled with GC–MS Analysis

The fermentation broths collected on day 7 from the C3 and Blank groups were analyzed by HS-SPME–GC–MS for volatile profiling, with three biological replicates per group. The HS-SPME–GC–MS analysis was performed according to a previously reported method [31], with appropriate modifications. Each sample (5.0 mL) was transferred to a 40 mL headspace vial containing sodium chloride (2 g) and boiling water (10 mL), then magnetically stirred at 80 °C for 15 min. A DVB/CAR/PDMS SPME fiber, preconditioned at 230 °C for 50 min, was exposed to the headspace at 80 °C for 50 min, followed by thermal desorption in the GC injection port for 3 min.

Chromatographic separation was performed on an HP-INNOWax capillary column (60.0 m × 0.25 mm, 0.25 μm) with helium as carrier gas (1 mL/min). The oven temperature program was: 50 °C for 5 min, ramped to 150 °C at 5 °C/min and held for 10 min, then ramped to 230 °C at 5 °C/min and held for 10 min. Injection was conducted in splitless mode (1 μL). MS parameters were: full-scan mode, electron energy 70 eV, ion source temperature 230 °C, detector voltage 350 V, emission current 0.20 mA, and detector temperature 250 °C.

### 2.7. Data and Statistical Analysis

Figures were generated using GraphPad Prism 9.5.1 (GraphPad Software, Boston, MA, USA) and OriginPro 2024 (OriginLab Corporation, Northampton, MA, USA). Data from the growth, physicochemical, and theaflavin-content assays, data were expressed as mean ± SD (*n* = 3). Differences among fermentation times were evaluated by one-way analysis of variance (ANOVA), followed by Tukey’s B post hoc test using IBM SPSS Statistics 30.0.0 (IBM Corp., Armonk, NY, USA). Comparisons between the C3 and Blank groups were performed using a two-tailed unpaired Student’s *t*-test in GraphPad Prism 9.5.1. Differences were considered statistically significant at *p* < 0.05. For GC–MS data processing, volatile compounds were putatively annotated by matching their mass spectra against standard spectral libraries. The relative abundance of each compound was estimated using peak-area normalization. Before pattern-recognition analysis, LC–MS data were processed with Progenesis QI v2.3 (Nonlinear Dynamics, Newcastle, UK). Data preprocessing involved baseline removal, peak picking and integration, retention-time adjustment, peak alignment, and signal normalization. Metabolite annotation was performed against the Human Metabolome Database (HMDB), LipidMaps version 2.3, and METLIN. The preprocessed qualitative and quantitative data matrix was then imported for multivariate statistical analyses, including principal component analysis (PCA) and orthogonal partial least-squares discriminant analysis (OPLS-DA). Differential metabolites were selected based on VIP values > 1 in the OPLS-DA model combined with *p* < 0.05 in univariate analysis. These differential metabolites were further submitted to the KEGG database for metabolic pathway enrichment analysis and pathway mapping.

## 3. Results and Discussion

### 3.1. Microbial Community Composition Difference in Theaflavin–Sucrose Kombucha

To compare the microbial community profiles of the black tea–sucrose and theaflavin–sucrose systems and to identify potential dominant responders, metagenomic analysis was performed on both fermentation systems. The results showed differences in the relative-abundances of the core microbial taxa between the black tea–sucrose and theaflavin–sucrose systems. At the genus level, both systems were dominated by *Komagataeibacter*, *Lodderomyces*, and *Meyerozyma*, but their relative abundance profiles differed between the two systems. In the black tea–sucrose system, Komagataeibacter was the predominant genus. In the theaflavin–sucrose sample, Komagataeibacter accounted for 21.56% of the annotated community, whereas *Lodderomyces* and *Meyerozyma* showed higher relative abundances than those observed in the black tea–sucrose sample (Figure 1A). The genus-level Circos plot further visualized the differences in the distribution of dominant taxa between the two systems, while the heatmap based on normalized relative abundance further illustrated the distinct genus-level abundance patterns observed in the BP and TF samples (Figure 1C,E). Overall, the microbial community profile of the theaflavin–sucrose system was characterized by reduced bacterial dominance and a greater relative representation of yeast taxa compared with the black tea–sucrose system. Previous studies have shown that acetic acid bacteria and yeasts usually constitute the core microbiota of kombucha, with their relative abundances being highly responsive to substrate composition and fermentation conditions [6,32,33,34]. Given the key roles of *Komagataeibacter* in organic acid production and cellulose biosynthesis, and the metabolic advantages of yeasts in sugar utilization and substrate transformation [35,36,37], the theaflavin-based substrate may have reduced the relative dominance of some acetic acid bacteria while favoring yeast taxa with stronger tolerance and metabolic adaptability [22,23,32].

Species-level profiling further resolved the taxonomic differences between the two fermentation systems. The dominant species in the black tea–sucrose system, *Komagataeibacter xylinus* and *Komagataeibacter europaeus*, decreased to 5.45% and 4.85%, respectively, in the theaflavin–sucrose system. Conversely, *Lodderomyces elongisporus* and *Meyerozyma guilliermondii* increased to 25.29% and 20.92%, respectively (Figure 1B). Consistently, the species-level Circos plot highlighted the greater relative contribution of yeast species in the TF system, and the corresponding heatmap separated the dominant species into distinct abundance patterns between the BP and TF systems (Figure 1D,F). These results suggest that, under theaflavin-enriched conditions, yeast taxa may play a more prominent role in substrate utilization and metabolic transformation within the fermentation system. In particular, the pronounced enrichment of *M. guilliermondii*, together with the reported environmental adaptability, broad substrate-utilization capacity, and potential for xenobiotic transformation within this genus [38,39], suggests that this species may be a candidate functional microorganism involved in the microbial response to theaflavin.

### 3.2. Functional Annotation of the Kombucha Microbiome

KEGG pathway annotation showed that, although the two fermentation systems shared broadly similar pathway categories, carbohydrate metabolism-related functions were more highly represented in the theaflavin group (Figure 2A,B). These functional differences suggested a shift in the metabolic potential of the kombucha microbiome in the theaflavin–sucrose system. The total number of annotated non-redundant genes was higher in the theaflavin sample (14,929 genes) than in the black-tea control (11,749 genes) (Supplementary Table S2). SEED hierarchical annotation further supported this functional shift, showing a greater representation of genes related to amino acid and carbohydrate metabolism in the theaflavin system. Specifically, 830 and 1375 genes were assigned to amino acids and derivatives and carbohydrate metabolism, respectively, in the TF system, compared with 467 and 663 genes in the BP system (Supplementary Table S3). These results suggest that the theaflavin–sucrose system had a greater representation of functions related to carbohydrate metabolism and substrate utilization. Previous studies have shown that carbohydrate metabolism, energy metabolism, and amino acid metabolism are among the core functional categories in kombucha and can vary according to substrate composition and fermentation stage [32,40]. Moreover, complex polyphenol-rich environments may induce metabolic reprogramming in yeasts, redirecting metabolic resources toward substrate transformation [22]. From the perspective of microbial community adaptation, increased environmental metabolic complexity can drive communities toward greater dependence on complex-substrate degradation and cross-feeding interactions [41,42].

CAZy annotation revealed glycoside hydrolases (GHs) and glycosyltransferases (GTs) as the predominant carbohydrate-active enzyme classes in both systems. It further identified 208 GH- and 239 GT-associated genes in the TF system, compared with 143 and 192, respectively, in the BP system (Figure 2C,D). CAZymes mainly participate in the degradation, modification, and formation of glycosidic bonds, and their representation provides insight into the carbohydrate-utilization potential of microbial communities [43]. The greater representation of GH- and GT-associated genes in the TF system suggests greater potential for glycosidic-bond hydrolysis and glycosyl transfer reactions, consistent with the subsequent metabolomic changes in oligosaccharides and selected glycosylated flavonoid/flavane compounds. Previous studies have shown that glycoside hydrolases and glycosyltransferases can mediate the glycosylation or transglycosylation of flavonoids [43,44,45]. In addition, theaflavins possess a benzotropolone skeleton, and their microbial transformation may also involve degalloylation, oxidation, and the formation of low-molecular-weight phenolic acids [18,46]. The CAZy profile supports greater carbohydrate metabolic potential in the theaflavin–sucrose system and may provide an enzymatic basis for the structural modification of polyphenol-derived metabolites. Whether CAZymes directly contribute to polyphenol structural modification warrants further enzymatic characterization.

### 3.3. Growth, Acidification, and Theaflavin Changes in Strain C3 in the Theaflavin Fermentation System

Based on preliminary isolation and screening results, strains C3 and C5 were identified as *Meyerozyma* sp. and *Lodderomyces elongisporus*, respectively, both of which were among the dominant yeast taxa enriched in the theaflavin-based substrate. However, C3 showed a stronger capacity for tea polyphenol transformation than C5 [24]. In addition, to avoid potential interference from lipid-accumulation-related side metabolism of C5 under high-sugar conditions [47], *Meyerozyma* sp. C3 was selected for subsequent experiments. During the 15-day cultivation in the theaflavin–sucrose system, strain C3 showed an overall increase in OD600 (Figure 3A). Continuous cell growth was observed during the early fermentation stage, 1–7 d, followed by a stationary phase from day 9 to day 15. The final OD600 reached 0.335 on day 15. Yeasts of the genus *Meyerozyma* have broad substrate-utilization capacities and can maintain stable metabolic activity in diverse complex matrices [38], which is consistent with the sustained growth of C3 in the theaflavin-based system. The fermentation system underwent progressively acidified as C3 cultivation. The pH of the C3 group decreased continuously after inoculation and reached 4.03 on day 15, whereas the pH of the control group remained largely stable (Figure 3B). Total acidity also increased with fermentation time and reached 0.316 g/L on day 15 (Figure 3C). Previous studies have shown that yeast strains alone can induce acid accumulation and pH reduction in fermentation systems, although acid-producing capacity differs among strains [23,37]. In this study, the accumulation of organic acids was consistent with the significant upregulation of citric acid and isocitric acid detected by metabolomics, indicating that strain C3 retained stable acid-producing capacity even in the absence of acetic acid bacteria. Theaflavins can undergo microbial transformations through processes such as degalloylation and C-ring cleavage, generating a series of low-molecular-weight phenolic metabolites [48]. On day 7, the total theaflavin content of the C3 group was significantly reduced compared with the control group (*p* < 0.05) (Figure 3D), with a conversion rate of 39.5%. These results demonstrate that strain C3 was capable of biotransforming theaflavins, and the resulting transformation products were further characterized by metabolomic analysis.

### 3.4. LC–MS-Based Untargeted Metabolomic Analysis

#### 3.4.1. Qualitative Annotation and Multivariate Analysis of Fermentation Metabolites

A total of 3237 metabolites were annotated in positive and negative ion modes, among which 1906 were assigned to defined chemical classes. The annotated metabolites mainly included lipids and lipid-like molecules (*n* = 717), organooxygen compounds (*n* = 273), organic acids and derivatives (*n* = 237), and phenylpropanoids and polyketides (*n* = 217). This broad metabolite distribution indicates that the metabolic products generated by strain C3 in the theaflavin–sucrose system are associated with multiple metabolic processes, including carbohydrate metabolism, organic acid metabolism, and polyphenol-related transformation, thereby providing a basis for subsequent screening and functional interpretation of differential metabolites.

To evaluate metabolic differences between the C3 and Blank groups, multivariate statistical analyses were performed. PCA showed that PC1 and PC2 collectively accounted for 56.3% of the total variance. The three biological replicates of the C3 group clustered on the negative axis of PC1, whereas those of the Blank group were distributed on the positive axis, indicating clear separation between the two groups (Figure 3E). PLS-DA and OPLS-DA further confirmed this grouping pattern (Figure 3F,G). The model parameters, R^2^X = 0.862 and Q^2^ = 0.988 (Figure 3H), indicated good model fitting and predictive performance. Collectively, these findings demonstrate that the metabolite profiles differed systematically between the two groups and that C3 fermentation substantially reshaped the metabolic composition of the theaflavin–sucrose system.

#### 3.4.2. Differential Metabolite Analysis

The volcano plot integrated fold change, statistical significance, and VIP values to visualize the overall distribution of differential metabolic features between the C3-fermented and unfermented groups (Figure 4A). Using VIP > 1 and *p* < 0.05 as screening criteria, 228 differential metabolite features were detected, including 171 upregulated and 57 downregulated features, accounting for 75% and 25% of the total, respectively. Among them, 99 metabolites were identified and further classified by chemical class (Figure 4B and Supplementary Tables S4–S7). Hierarchical clustering of the top 50 differential metabolites further confirmed the systematic divergence in metabolite profiles between the two groups (Figure 4C). Among the annotated differential metabolites, 30 were carbohydrates and derivatives, mainly oligosaccharides (Table 1). After fermentation, the contents of several oligosaccharides, including trehalose, maltotriose, fructan, cellotetraose, 1-kestose, and raffinose, increased markedly (*p* ≤ 0.01). When sucrose served as the primary carbon source, the accumulation of multiple oligosaccharides after C3 fermentation was probably associated with sucrose hydrolysis and transglycosylation-related enzymatic activities. It has been reported that microbial fructosyltransferases and β-fructofuranosidases can catalyze the conversion of sucrose into fructooligosaccharides and related oligosaccharides [49,50]. Trehalose accumulation in yeasts is commonly associated with adaptive responses to osmotic, acidic, and other stress conditions, and may therefore reflect the stress response through which C3 maintained cellular homeostasis in the polyphenol-rich matrix [51]. Many of these oligosaccharides possess prebiotic potential by selectively promoting beneficial intestinal bacteria and short-chain fatty acid production, while trehalose has also been reported to exert cytoprotective and metabolic regulatory effects [51,52,53,54].

Analysis of flavonoid and flavan metabolites provides insight into the potential degradation and biotransformation products of theaflavins during fermentation. After 7 d of fermentation by strain C3, 15 differential flavonoid/flavan compounds were detected in the fermentation broth (Table 2). Compared with the unfermented samples, three catechins, including epigallocatechin gallate (EGCG), (+)-gallocatechin (GC), and (-)-epigallocatechin (EGC), and five catechin-derived compounds, including gallocatechin 3′-O-gallate, (-)-epigallocatechin 3-(4-methyl-gallate), (-)-epigallocatechin 3-(3-methyl-gallate), epigallocatechin 3,5-digallate, and theasinensin F, increased markedly. The remaining compounds were mainly methylated, methoxylated, glycosylated, or sulfated flavonoid derivatives. In particular, rhamnazin 3-O-sulfate represented a sulfated flavonoid metabolite, whereas luteolin 7-(6″-p-benzoyglucoside), kaempferide 3-rhamnoside-7-(6″-succinylglucoside), and 6-methoxykaempferol 3,7-bis(3-acetylrhamnoside) were glycosylated or acylated glycoside derivatives. This metabolite pattern suggests that strain C3 participated in the metabolic transformation and secondary structural modification of theaflavin-related polyphenol structures during fermentation. Microorganisms can cleave galloyl ester bonds in theaflavins through tannase and other hydrolases, releasing catechin nuclei and gallic acid, which can subsequently be converted into structurally diverse derivatives through glycosylation, methylation, and related enzymatic reactions [18,55]. This pattern is similar to the metabolic profile observed in EGCG liquid-state fermentation mediated by *Eurotium cristatum* [56]. Notably, theasinensin F was also detected as an upregulated differential metabolite after fermentation. As an oxidative condensation dimer of catechin units, theasinensins are formed through oxidative coupling of catechin B-ring ortho-quinones under enzymatic or non-enzymatic conditions during tea processing [57,58]. Its accumulation in the present fermentation system therefore indicates that oxidative remodeling of polyphenol structures occurred in parallel with hydrolytic degradation, suggesting that the metabolic transformation of theaflavins by strain C3 is not limited to a single degradative pathway. Fourteen differential metabolites were organic acids and derivatives, represented by isocitric acid, citric acid, and quinic acid (Supplemental Table S4). Isocitric acid and citric acid are both involved in the tricarboxylic acid cycle, which is consistent with the continuous acidification and total acid accumulation observed in the C3 fermentation system.

### 3.5. Metabolic Pathway Analysis

KEGG enrichment analysis mapped 17 differential metabolites to 19 metabolic pathways. Among these, galactose metabolism, the citrate cycle, starch and sucrose metabolism, and glyoxylate and dicarboxylate metabolism met the enrichment criteria (Figure 4D). In the galactose metabolism pathway, raffinose, D-tagatose, and sucrose were the main enriched metabolites. In yeasts, raffinose can be hydrolyzed by α-galactosidase to release galactose, which is further metabolized through the Leloir pathway [59,60]. D-tagatose, a C4 epimer of galactose, was enriched together with raffinose in the galactose metabolism pathway, suggesting that its accumulation may be associated with activation of this pathway; however, the specific conversion mechanism requires further investigation. The simultaneous enrichment of these metabolites indicates that strain C3 activated galactose-related metabolic branches even when sucrose was the major carbon source. Several galactosylated flavonol glycosides have been detected in tea [61], and galactose residues released from their hydrolysis may represent one potential source for activating this metabolic branch. Nevertheless, their specific origin in the present theaflavin system remains to be clarified.

Citric acid and isocitric acid were simultaneously enriched in the citrate cycle and glyoxylate and dicarboxylate metabolism pathways. Both metabolites are key intermediates of the tricarboxylic acid cycle. In some yeasts, downstream reactions of the TCA cycle can be partially restricted under nitrogen-limited conditions, resulting in citric acid and isocitric acid accumulation [62,63]. Therefore, the accumulation of these two metabolites suggests that the TCA-cycle metabolic flux of strain C3 may have been partially constrained during the middle and late stages of fermentation. In the starch and sucrose metabolism pathway, dextrin and sucrose were both upregulated and enriched, indicating that sucrose utilization by strain C3 was not exhaustive. Sucrose resynthesis or repolymerization of partially hydrolyzed products may have occurred during fermentation, suggesting a degree of metabolic flexibility in carbon-source management. Overall, the systematic enrichment of these pathways indicates that strain C3 fermentation activated two major metabolic directions, carbohydrate transformation and organic acid accumulation, thereby providing a pathway-level explanation for the acidification phenotype and the formation of diverse fermentation metabolites.

### 3.6. HS-SPME–GC–MS Analysis of Volatile Compounds

After 7 d of fermentation by strain C3, 29 volatile compounds were detected across the fermented and unfermented groups, including 8 esters, 6 ketones, 5 alcohols, 4 phenols, 4 aldehydes, and 2 acids. Seventeen compounds were detected in the unfermented control, whereas 22 were detected after fermentation. Thirteen volatile compounds were newly detected in the C3-fermented group, while the relative contents of 11 compounds decreased after fermentation. Among the compounds that increased after fermentation, 2-phenylethanol was the most prominent, reaching a relative content of 19.69% (Table 3). As a characteristic yeast-derived aroma compound, 2-phenylethanol is produced from L-phenylalanine via the Ehrlich pathway and contributes a pleasant rose-like aroma [64,65]. Its marked accumulation is consistent with active aromatic amino acid catabolism by strain C3 during fermentation. 4-Ethylphenol, with a relative content of 4.2103%, was newly detected in the fermented group. This compound can be formed from p-coumaric acid through sequential reactions catalyzed by hydroxycinnamate decarboxylase and vinylphenol reductase [66]. Its formation suggests that polyphenol-derived intermediates may contribute to the volatile profile of the fermented theaflavin–sucrose system. By contrast, several substrate-derived volatiles decreased or were no longer detected after fermentation, including 6-methyl-5-hepten-2-one and decanal, also known as decyl aldehyde. This reduction may be associated with yeast-mediated conversion of aldehydes and ketones, as alcohol dehydrogenases can reduce carbonyl compounds to their corresponding alcohols [67]. Several phthalate esters were also detected in the GC–MS profiles (Table 3). Dibutyl phthalate and diisobutyl phthalate have previously been reported in kombucha volatile profiles [68], although phthalates are also recognized as common background contaminants in GC–MS analyses [69]. Accordingly, these signals were not used to infer C3-associated aroma formation, and the interpretation of fermentation-related volatile changes focused on compounds with clearer links to fermentation metabolism, including 2-phenylethanol and 4-ethylphenol. These fermentation-derived volatiles, together with the restructured metabolite profile revealed by LC–MS analysis, suggest that strain C3-mediated fermentation may substantially alter the sensory characteristics of the theaflavin–sucrose system. While the present study focused on elucidating microbial mechanisms and metabolic pathways through multi-omics approaches, future investigations incorporating comprehensive sensory evaluation and consumer acceptance testing would provide valuable insights into the organoleptic quality and market potential of theaflavin-fermented beverages.

### 3.7. Evaluation of Techno-Functional and Biosafety Potential

Strain C3 used in this study was previously isolated from kombucha and identified as *Meyerozyma* sp. C3 was identified based on ITS sequence analysis, showing 99.29% similarity to *M. guilliermondii* (accession no. NR111247) [24]. Given its close phylogenetic relationship, the reported functional characteristics of *M. guilliermondii* provide important references for evaluating strain C3. *M. guilliermondii* is a non-conventional yeast recognized for its robust environmental adaptability, broad substrate utilization capacity, and biotransformation potential [38]. This species has been isolated from diverse fermented foods and beverages, including kombucha, wine, and traditional fermented vegetables [70,71,72]. Several *M. guilliermondii* strains are capable of producing volatile compounds such as esters, higher alcohols, and terpenes, thereby influencing the aroma and flavor profiles of fermented products [40,41]. Additionally, some strains exhibit β-glucosidase activity, enabling the release of glycoside-bound aroma precursors and the hydrolysis of phenolic glycosides such as isoflavone glucosides [39,73]. From a biosafety perspective, *M. guilliermondii* has demonstrated a well-established safety record in food fermentation applications. This species is generally not considered a human pathogen, and multiple strain-level safety assessments support its application potential in food systems. Safety evaluation of *M. guilliermondii* strains intended for food fermentation typically focuses on key indicators including antifungal drug susceptibility, virulence-associated phenotypes (e.g., gelatinase, DNase, hemolysin production), and biogenic amine-producing capacity. Current evidence indicates that systematically evaluated *M. guilliermondii* strains generally do not exhibit pathogenicity-related characteristics, do not produce significant virulence factors, and maintain biogenic amine production below food safety thresholds [74,75]. Furthermore, U.S. regulations explicitly permit the use of specific strains derived from *C. guilliermondii* ATCC 20474 for citric acid fermentation production under defined conditions, further confirming the acceptability of this species in food industrial applications [76]. Combined with the context that *M. guilliermondii* has been identified as a member of kombucha microbial communities and investigated for probiotic properties [70], this body of evidence collectively supports the biosafety foundation of this species for use as a food fermentation agent.

In the present study, strain C3, originating from a traditional kombucha fermentation system, demonstrated stable growth, sustained acidification, and effective theaflavin biotransformation in a defined theaflavin-containing fermentation system. These fermentation characteristics are consistent with the reported functional performance of *M. guilliermondii*. Based on its high phylogenetic similarity to *M. guilliermondii* and the established safety profile of this species, strain C3 exhibits promising potential for functional fermented tea beverage development. To ensure safety for industrial-scale application, future work will conduct systematic strain-specific safety assessments of strain C3, including antifungal drug susceptibility testing, virulence-associated phenotype screening, and biogenic amine production capacity evaluation.

## 4. Conclusions

This study investigated the fermentation performance and theaflavin-biotransformation capacity of *Meyerozyma* sp. C3 in a simplified theaflavin–sucrose system. Strain C3 grew stably in the theaflavin matrix, continuously acidified the medium, and achieved a theaflavin conversion rate of 39.5% within 7 days, demonstrating its capacity for the biotransformation of theaflavins. Integrated metagenomic and metabolomic evidence indicated that theaflavins were converted into structurally diverse catechin-related derivatives, potentially involving hydrolysis, glycosylation, and other secondary modifications. In parallel, C3 fermentation was associated carbohydrate metabolism and tricarboxylic acid cycle-associated pathways, leading to the accumulation of oligosaccharides with potential prebiotic relevance, organic acids, and a characteristic volatile profile dominated by 2-phenylethanol. Collectively, these findings demonstrate the potential of *Meyerozyma* sp. C3 as a promising functional yeast for directing theaflavin transformation and flavor formation in tea-based fermented products, and provide both a strain resource and a mechanistic framework for optimizing polyphenol composition and enhancing the functional quality of kombucha and related fermented tea beverages. It should be noted that the community-level metagenomic analysis was based on pooled fermentation samples, and the proposed transformation routes were inferred primarily from integrated functional and metabolomic profiles. Future studies incorporating independently sequenced fermentation replicates and targeted validation of key enzymes and transformation intermediates will further resolve the molecular mechanisms underlying theaflavin biotransformation by strain C3 and facilitate its industrial application in tea-based fermentation.

## Figures and Tables

**Figure 1:**
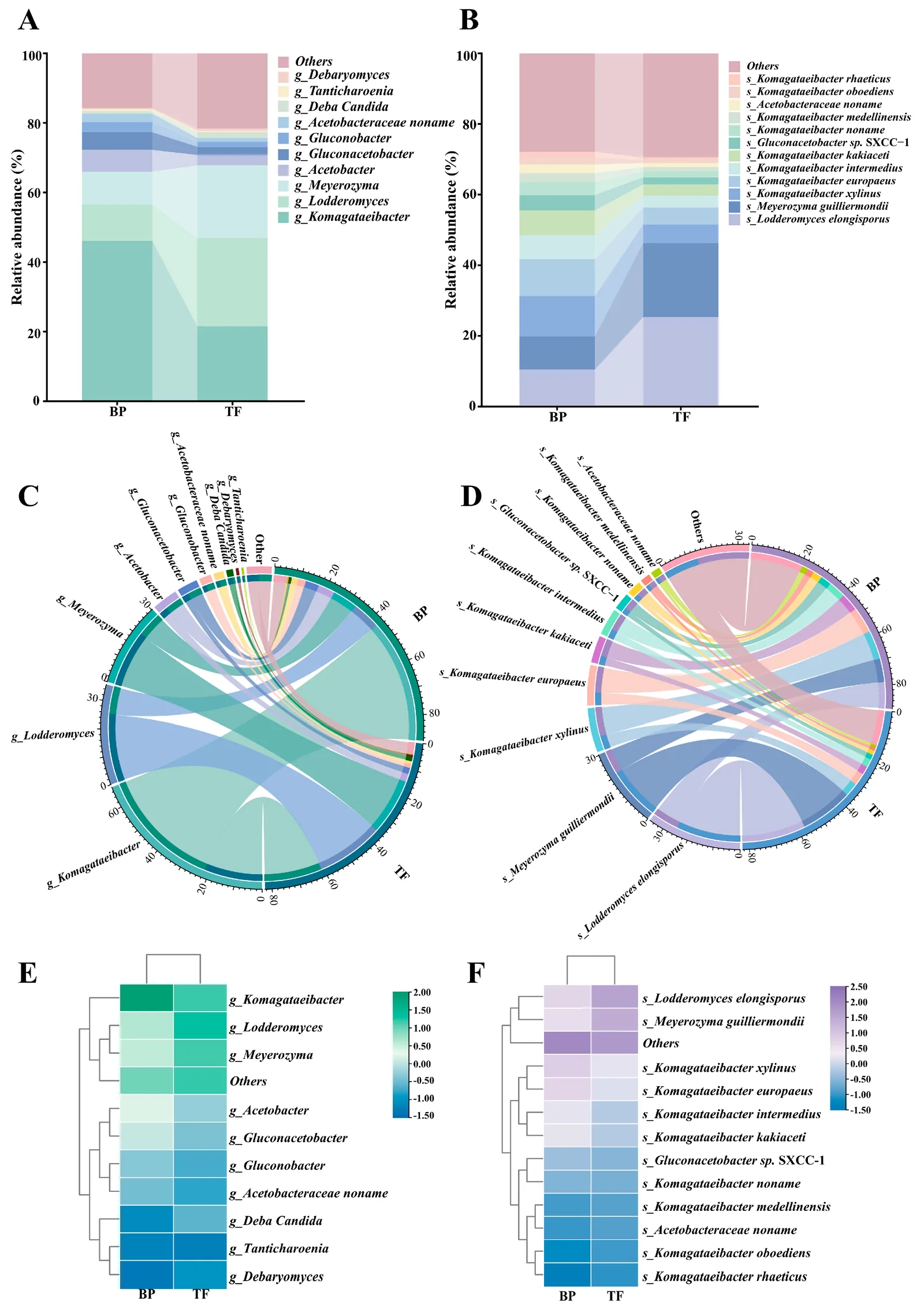
Metagenomic profiling of microbial community composition in the black tea–sucrose (BP) and theaflavin–sucrose (TF) systems. (**A**,**B**) Relative abundance of dominant taxa at the genus (**A**) and species (**B**) levels. (**C**,**D**) Circos plots showing the distribution of dominant genera (**C**) and species (**D**) between the two systems. (**E**,**F**) Heatmaps of the dominant genera (**E**) and species (**F**) based on normalized relative abundance. BP, black tea–sucrose system; TF, theaflavin–sucrose system. Low-abundance taxa were grouped as “Others”.

**Figure 2:**
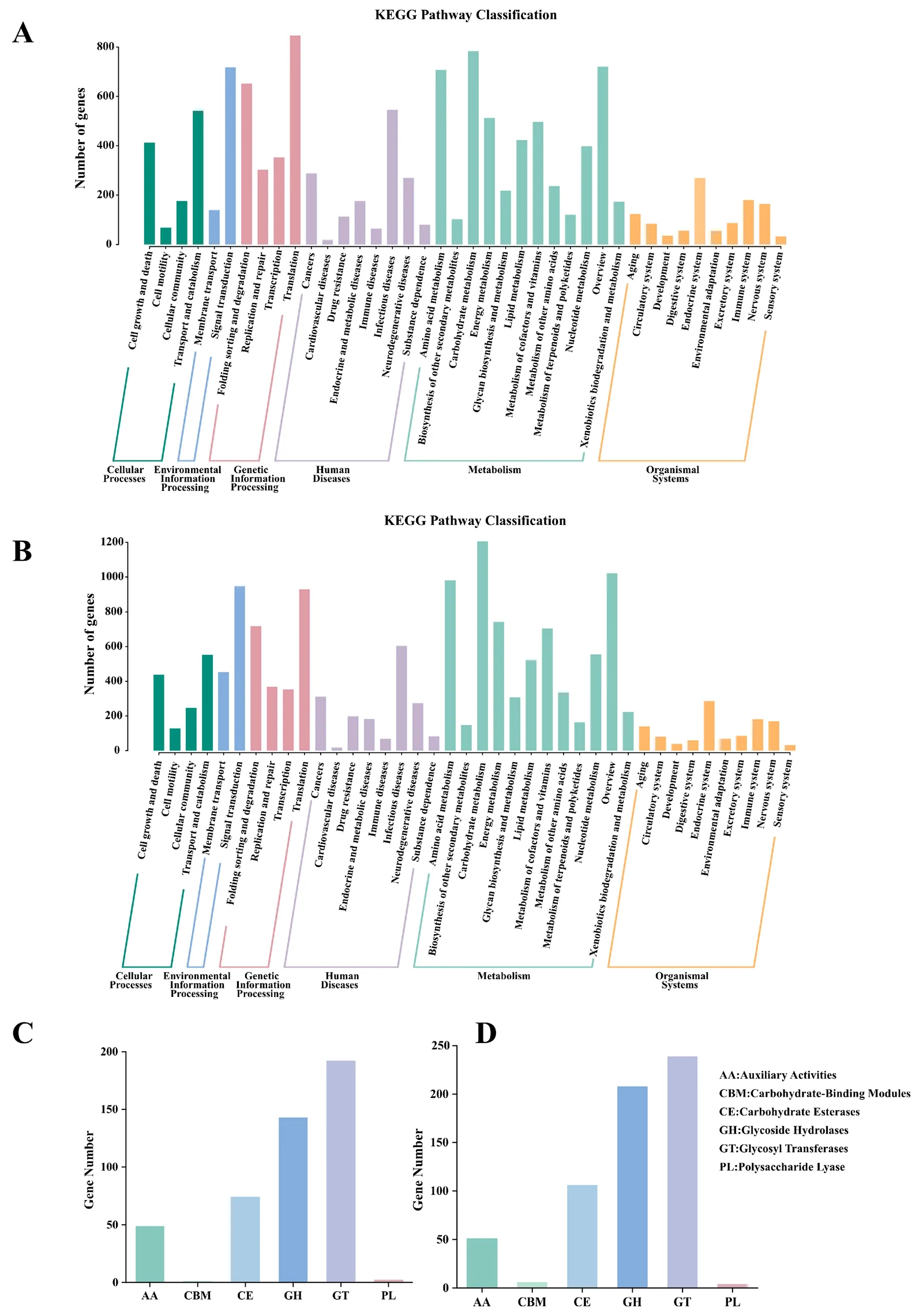
Functional annotation of the kombucha microbiome in the BP and TF systems. (**A**,**B**) KEGG pathway classification of annotated genes in the BP (**A**) and TF (**B**) systems. (**C**,**D**) CAZy classification of carbohydrate-active enzyme genes in the BP (**C**) and TF (**D**) systems. Bar heights indicate the number of annotated genes assigned to each functional category.

**Figure 3:**
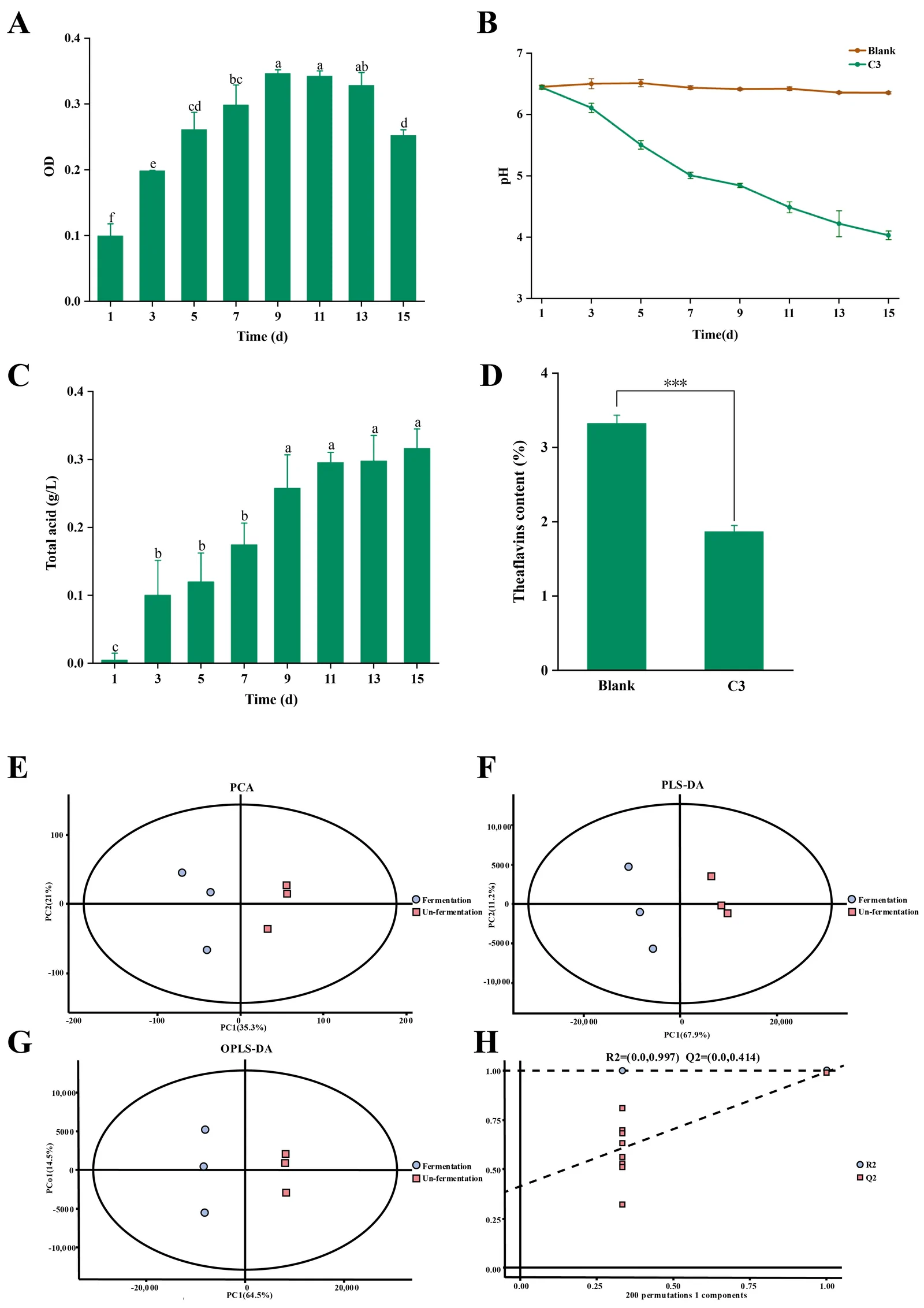
Fermentation characteristics of strain C3 (*Meyerozyma* sp. C3) in the theaflavin–sucrose system and LC–MS-based multivariate analysis. (**A**) Growth dynamics of strain C3 during fermentation, expressed as OD_600_. (**B**) pH changes in the C3-fermented and uninoculated control (Blank, groups). (**C**) Changes in total acidity during 15 d of fermentation. (**D**) Total theaflavin content in the C3 and Blank groups on day 7. (**E**–**G**) PCA, PLS-DA, and OPLS-DA score plots of the C3-fermented and unfermented samples. (**H**) Permutation validation of the OPLS-DA model. Data are expressed as mean ± SD, *n* = 3. Different lowercase letters indicate significant differences among fermentation times, *p* < 0.05. Asterisks indicate significant differences between the C3 and Blank groups, *** *p* < 0.001.

**Figure 4:**
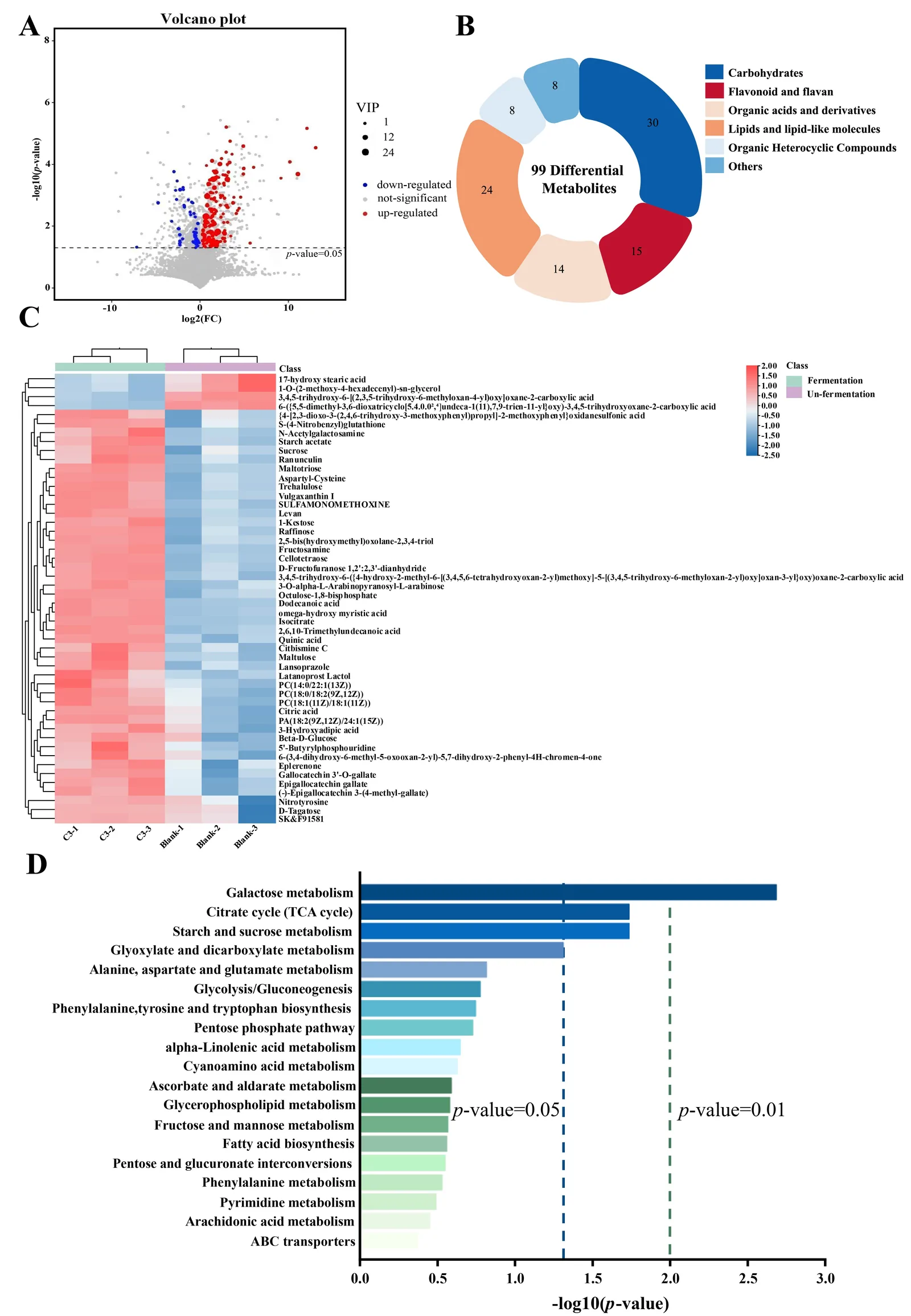
Differential metabolite profiling and pathway enrichment after fermentation by strain C3 (*Meyerozyma* sp. C3). (**A**) Volcano plot of differential metabolites between the C3-fermented and unfermented groups. Red and blue dots indicate up- and down-regulated metabolites, respectively, and grey dots indicate non-significant features. Dot size represents the VIP value. (**B**) Relative distribution of the 99 putatively annotated differential metabolites among major chemical classes. (**C**) Hierarchical clustering heatmap of the top 50 differential metabolites ranked by the degree of difference. Differential metabolites were selected using VIP > 1 and *p* < 0.05. Peak areas were log-transformed and row-scaled by Z-score normalization. Colors indicate relative abundance from low to high. C3-1, C3-2, and C3-3 represent the three biological replicates of the C3 group, whereas Blank-1, Blank-2, and Blank-3 represent the three biological replicates of the Blank group. (**D**) KEGG enrichment analysis of the top 20 metabolic pathways differing between the C3-fermented and unfermented groups. Dashed lines indicate *p* = 0.05 and *p* = 0.01.

**Table 1:** Differential carbohydrate metabolites.

*m*/*z*	Retention Time	Metabolites	Molecular Formula	VIP	*p* Value	FC	log_2_(FC)
387.114	0.85	Maltulose	C_12_H_22_O_11_	33.70	0.005	1.59	0.67
342.139	0.85	D-Fructofuranose 1,2′:2,3′-dianhydride	C_12_H_20_O_10_	24.94	0.000	2.84	1.51
360.150	0.86	Trehalulose	C_12_H_22_O_11_	23.26	0.001	1.76	0.81
549.167	0.82	Maltotriose	C_18_H_32_O_16_	17.27	0.000	8.55	3.10
522.203	0.81	Levan	C_18_H_32_O_16_	16.56	0.000	3.38	1.76
225.061	0.76	Beta-D-Glucose	C_6_H_12_O_6_	16.55	0.041	3.58	1.84
198.097	2.01	D-Tagatose	C_6_H_12_O_6_	14.26	0.033	2.47	1.31
180.087	0.78	Fructosamine	C_6_H_13_NO_5_	9.63	0.000	4.76	2.25
203.052	0.78	2,5-bis(hydroxymethyl)oxolane-2,3,4-triol	C_6_H_12_O_6_	9.47	0.000	1.97	0.98
365.105	0.90	Sucrose	C_12_H_22_O_11_	7.46	0.018	1.26	0.34
684.254	0.85	Cellotetraose	C_24_H_42_O_21_	6.24	0.000	2.58	1.37
666.244	0.85	A	C_24_H_40_O_20_	5.90	0.000	3.38	1.75
527.158	0.81	1-Kestose	C_18_H_32_O_16_	5.46	0.001	2.96	1.56
845.278	0.82	3-O-alpha-L-Arabinopyranosyl-L-arabinose	C_30_H_54_O_27_	4.88	0.002	13.31	3.73
358.134	0.78	B	C_12_H_20_O_11_	4.84	0.001	0.21	−2.25
527.158	1.13	Raffinose	C_18_H_32_O_16_	4.77	0.000	10.07	3.33
204.086	0.78	N-Acetylgalactosamine	C_8_H_15_NO_6_	3.53	0.018	3.26	1.70
391.101	0.78	C	C_17_H_20_O_9_	2.86	0.002	0.04	−4.74
438.980	1.13	Octulose-1,8-bisphosphate	C_8_H_18_O_14_P_2_	2.84	0.000	8686.58	13.08
259.081	0.85	Ranunculin	C_11_H_16_O_8_	2.66	0.009	1.40	0.49
505.176	0.85	Dextrin	C_18_H_32_O_16_	1.80	0.000	1.89	0.92
475.130	0.87	3,4,5-trihydroxy-6-(2-hydroxyethoxy)oxane-2-carboxylic acid	C_8_H_14_O_8_	1.69	0.000	5.41	−2.44
339.093	0.78	Lactobionic acid	C_12_H_22_O_12_	1.50	0.016	0.62	−0.70
384.150	0.78	Beta-1,4-mannose-N-acetylglucosamine	C_14_H_25_NO_11_	1.49	0.000	6.04	−0.70
723.185	0.91	Galabiose	C_24_H_44_O_22_	1.46	0.005	0.68	0.55
535.151	0.85	1,4-beta-D-Glucan	C_18_H_32_O_18_	1.19	0.032	0.58	−0.79
387.094	0.89	Glucocaffeic acid	C_15_H_18_O_9_	1.13	0.002	7.52	2.91
437.126	1.13	beta-D-Xylopyranosyl-(1->5)-alpha-L-arabinofuranosyl-(1->3)-L-arabinose	C_15_H_26_O_13_	1.13	0.002	9.10	3.19
173.008	1.13	Diketogulonic acid	C_6_H_8_O_7_	1.05	0.000	12.34	3.63
745.141	4.65	Sennoside monoglucoside B	C_36_H_28_O_15_	1.03	0.000	2.84	1.50

Note: A. 3,4,5-trihydroxy-6-({4-hydroxy-2-methyl-6-[(3,4,5,6-tetrahydroxyoxan-2-yl)methoxy]-5-[(3,4,5-trihydroxy-6-methyloxan-2-yl)oxy]oxan-3-yl}oxy)oxane-2-carboxylic acid; B. 3,4,5-trihydroxy-6-[(2,3,5-trihydroxy-6-methyloxan-4-yl)oxy]oxane-2-carboxylic acid; C. 6-({5,5-dimethyl-3,6-dioxatricyclo [5.4.0.0^2^,^4^]undeca-1(11),7,9-trien-11-yl}oxy)-3,4,5-trihydroxyoxane-2-carboxylic acid.

**Table 2:** Differential flavonoid and flavan metabolites.

*m*/*z*	Retention Time	Metabolites	Molecular Formula	VIP	*p* Value	FC	log_2_(FC)
457.078	4.79	Epigallocatechin gallate	C_22_H_18_O_11_	4.64	0.005	2.63	1.40
459.092	4.80	Gallocatechin 3′-O-gallate	C_22_H_18_O_11_	3.17	0.003	2.18	1.13
379.082	0.87	6-(3,4-dihydroxy-6-methyl-5-oxooxan-2-yl)-5,7-dihydroxy-2-phenyl-4H-chromen-4-one	C_21_H_18_O_8_	3.14	0.038	1.33	0.41
471.093	5.11	(-)-Epigallocatechin 3-(4-methyl-gallate)	C_23_H_20_O_11_	2.17	0.005	2.59	1.37
271.060	4.95	2-(3,4,5-trihydroxyphenyl)-4H-chromen-4-one	C_15_H_10_O_5_	1.86	0.000	2.16	1.11
473.108	5.09	(-)-Epigallocatechin 3-(3-methyl-gallate)	C_23_H_20_O_11_	1.73	0.001	2.41	1.27
570.160	5.17	Luteolin 7-(6″-p-benzoyglucoside)	C_28_H_24_O_12_	1.57	0.005	1.87	0.90
433.019	0.78	Rhamnazin 3-O-sulfate	C_17_H_14_O_10_S	1.42	0.003	3.46	1.79
375.070	0.78	Jaceosidin	C_17_H_14_O_7_	1.29	0.001	0.27	−1.88
747.154	4.64	Kaempferide 3-rhamnoside-7-(6″-succinylglucoside)	C_32_H_36_O_18_	1.28	0.000	3.68	1.88
289.071	4.80	(+)-Gallocatechin	C_15_H_14_O_7_	1.14	0.008	1.91	0.93
731.159	5.24	6-Methoxykaempferol 3,7-bis(3-acetylrhamnoside)	C_32_H_36_O_17_	1.13	0.001	3.12	1.64
609.088	5.11	Epigallocatechin 3,5-digallate	C_29_H_22_O_15_	1.12	0.008	2.84	1.51
899.163	5.15	Theasinensin F	C_44_H_34_O_21_	1.08	0.000	4.33	2.12
305.067	4.33	(-)-Epigallocatechin	C_15_H_14_O_7_	1.05	0.033	1.79	0.84

**Table 3:** Volatile compounds in the unfermented and C3-fermented samples.

Metabolites	Molecular Formula	Unfermentation		Fermentation	
		Comparability	Relative Content	Comparability	Relative Content
Menthol	C_10_H_20_O	72	0.3271	74	0.4365
2-Ethylhexanol	C_8_H_18_O	90	3.4502	-	-
2-Phenylethanol	C_8_H_10_O	-	-	93	19.6882
3,4-Dimethylbenzyl alcohol	C_9_H_12_O	-	-	96	0.6286
Alpha-Terpineol	C_10_H_18_O	-	-	86	0.3006
3,4-Dimethylbenzaldehyde	C_9_H_10_O	95	2.9701	-	-
Decanal	C_10_H_20_O	91	1.5582	-	-
3,5-Dimethylbenzaldehyde	C_9_H_10_O	-	-	93	16.2431
2,4-Dimethylbenzaldehyde	C_9_H_10_O	-	-	81	0.6665
2-Camphanone	C_10_H_16_O	95	0.1791	-	-
Methyl Heptenone	C_8_H_14_O	74	8.0344		
Acetophenone	C_8_H_8_O	87	0.1815	81	0.1315
Geranylacetone	C_13_H_22_O	94	0.5413	87	0.3287
(1S,4S)-1,7,7-Trimethylbicyclo [2.2.1]heptan-2-one	C_10_H_16_O	-	-	70	0.1262
Germacrone	C_13_H_18_O			81	0.1031
3-Hydroxy-2,2,4-trimethylpentyl isobutyrate	C_12_H_24_O_3_	74	0.2956	-	-
2,2,4-Trimethyl-1,3-pentanediol diisobutyrate	C_16_H_30_O_4_	-	-	72	0.296
Dibutyl Phthalate	C_16_H_22_O_4_	97	7.9981	96	2.4376
Dimethyl Phthalate	C_10_H_10_O_4_	97	0.1797	96	0.3351
Diethyl Phthalate	C_12_H_14_O_4_	93	0.1473	97	0.146
Diethylene Glycol Butyl Ether Acetate	C_10_H_20_O_4_	91	0.5492	91	0.2868
Dibutyl Maleate	C_12_H_20_O_4_	-	-	72	0.104
Diisobutyl Phthalate	C_16_H_22_O_4_	-	-	91	0.2743
Nonanoic Acid	C_9_H_18_O_2_	-	-	93	0.393
Decanoic Acid	C_10_H_20_O_2_	93	0.329	96	0.378
2,4-Di-tert-butylphenol	C_14_H_22_O	-	-	97	0.5829
4-Ethylphenol	C_8_H_10_O	-	-	95	4.2103
2,6-Di-tert-butyl-p-cresol	C_15_H_24_O	89	0.1058	-	-
2,5-Di-tert-butylphenol	C_14_H_22_O	95	3.4592	-	-
2,6-Di-tert-butylbenzoquinone	C_14_H_20_O_2_	95	0.7275	97	0.4283

## Data Availability

The original contributions presented in this study are included in the article/Supplementary Material. Further inquiries can be directed to the corresponding author.

## Supplementary Materials

The following supporting information can be downloaded at: https://www.mdpi.com/article/10.3390/foods15193453/s1. Supplemental Table S1. Quality control statistics of metagenomic sequencing data. Supplemental Table S2. Top 10 KEGG pathways with the highest number of annotated genes. Supplemental Table S3. The top 10 classes of SEED function. Supplemental Table S4. Differential Organic acids and derivatives metabolites. Supplemental Table S5. Differential lipids and lipid-like molecule metabolites. Supplemental Table S6. Differential organic heterocyclic compound metabolites. Supplemental Table S7. Other differential metabolites. Supplementary Figure S1. Heatmap analysis of microbial community composition at the class and family levels. Supplementary Figure S2. GO functional classification of metagenomic genes in the BP and TF systems. Supplementary Figure S3. The photos of novel isolated *Meyerozyma* sp. C3 yeast. Supplementary Figure S4. Yeast separation process.
