# Supplementary material for: Polyphenol Bioconversion and Volatile Aroma Production by Meyerozyma sp. C3 Isolated from Kombucha in the Theaflavin–Sucrose Fermentation Model
Source: Foods. 2026 Sep 27;15(19):3453. doi: 10.3390/foods15193453 (PMC13663958; doi:10.3390/foods15193453)

Supplemental Table S1. Quality control statistics of metagenomic sequencing data.

Sample	Total Reads Count (×10^7)	Total Bases Count (×10^{10}bp)	GC Bases Count (×10^9bp)	GC Bases Ratio	Average Read Length (bp)
BP	9.99	1.47	7.85	53.31%	147.38
TF	8.53	1.26	5.76	45.94%	147.22

Supplemental Table S2. Top 10 KEGG pathways with the highest number of annotated genes.

number	Pathway	Gene annotation	
		BP	TF
1	Translation	846	929
2	Carbohydrate metabolism	783	1205
3	Overview	720	1022
4	Signal transduction	718	947
5	Amino acid metabolism	707	981
6	Folding sorting and degradation	652	718
7	Infectious diseases	546	605
8	Transport and catabolism	541	553
9	Energy metabolism	513	743
10	Metabolism of cofactors and vitamins	497	704
11	total number of genes	11748	14929

Supplemental Table S3. The top 10 classes of SEED function.

number	Function categories	Gene number	
		BP	TF
1	Total number of annotated genes	6011	10433
2	Protein Metabolism	832	1088
3	Carbohydrates	663	1375
4	Clustering-based subsystems	593	1060
5	RNA Metabolism	533	681
6	Amino Acids and Derivatives	467	830
7	Cofactors, Vitamins, Prosthetic Groups, Pig-	396	594
8	DNA Metabolism	269	453
9	Stress Response	258	472
10	Fatty Acids, Lipids, and Isoprenoids	237	354
11	Nucleosides and Nucleotides	231	380

Supplemental Table S4. Differential Organic acids and derivatives metabolites.

m/z	Retention time (min)	Metabolites	Formula	VIP	P-value	FC	log2(FC)
191.019	1.13	Isocitrate	$C_6H_8O_7$	9.39	0.000	2161.69	11.08
145.049	0.85	3-Hydroxyadipic acid	$C_6H_{10}O_5$	7.25	0.024	2.01	1.01
289.092	0.85	Starch acetate	$C_{12}H_{18}O_9$	5.88	0.002	1.77	0.82
215.016	1.13	Citric acid	$C_6H_8O_7$	4.12	2.584	29.75	4.90
265.023	2.09	Nitrotyrosine	$C_9H_{10}N_2O_5$	3.38	0.046	2.30	1.20
362.097	0.79	Vulgaxanthin I	$C_{14}H_{17}N_3O_7$	3.23	0.001	2.31	1.21
191.055	0.76	Quinic acid	$C_7H_{12}O_6$	3.05	8.307	1146.31	10.16
217.029	0.76	Aspartyl-Cysteine	$C_7H_{12}N_2O_5S$	2.85	0.000	2.17	1.11
451.030	0.76	{4-[2,3-dioxo-3-(2,4,6-trihydroxy-3-methoxy-phenyl)propyl]-2-methoxyphenyl}oxidanesulfonic acid	$C_{17}H_{16}O_{11}S$	2.43	0.017	2.36	1.24
223.060	0.78	2-Succinylbenzoate	$C_{11}H_{10}O_5$	1.51	0.015	2.61	1.39
176.972	0.20	Fosfomycin	$C_3H_7O_4P$	1.22	0.021	0.72	-0.47
685.434	15.35	Gluten exorphin C	$C_{29}H_{57}N_5O_{12}$	1.22	0.048	0.01	-7.16
168.065	0.83	Phenylglyoxylic acid	$C_8H_6O_3$	1.21	0.026	2.08	1.05
270.967	1.43	2-[3-(sulfooxy)phenyl]acetic acid	$C_8H_8O_6S$	1.18	0.030	2.75	1.46

Supplemental Table S5. Differential Lipids and lipid-like molecules metabolites.

m/z	Retention time (min)	Metabolites	Formula	VIP	P-value	FC	log2(FC)
786.598	13.15	PC(18:0/18:2(9Z,12Z))	$C_{44}H_{84}NO_8P$	30.84	0.015	4.02	2.01
318.300	8.89	17-hydroxy stearic acid	$C_{18}H_{36}O_3$	4.04	0.032	0.76	-0.40
415.211	10.21	Eplerenone	$C_{24}H_{30}O_6$	3.82	0.030	1.24	0.31
218.211	5.84	Dodecanoic acid	$C_{12}H_{24}O_2$	3.73	0.000	30.77	4.94
800.614	12.55	PA(18:2(9Z,12Z)/24:1(15Z))	$C_{45}H_{83}O_8P$	2.70	0.004	3.16	1.66
808.580	13.73	PC(18:1(11Z)/18:1(11Z))	$C_{44}H_{84}NO_8P$	2.60	0.014	4.12	2.04
832.607	11.23	PC(14:0/22:1(13Z))	$C_{44}H_{86}NO_8P$	2.57	0.027	1.73	0.79
246.243	7.27	2,6,10-Trimethylundecanoic acid	$C_{14}H_{28}O_2$	2.47	0.000	9.35	3.23
262.238	5.97	omega-hydroxy myristic acid	$C_{14}H_{28}O_3$	2.38	0.000	29.91	4.90
362.326	8.99	1-O-(2-methoxy-4-hexadecenyl)-sn-glycerol	$C_{20}H_{40}O_4$	2.20	0.028	0.72	-0.48
432.251	10.11	PGH2-EA	$C_{23}H_{39}NO_4$	2.11	0.004	17.13	4.10
838.629	12.63	PC(16:0/22:1(13Z))[U]	$C_{46}H_{90}NO_8P$	2.10	0.004	2.94	1.56
732.551	12.29	1,2-Dierucoyl-SN-Glycero-3-Phosphoethanolamine	$C_{40}H_{78}NO_8P$	1.99	0.016	6.23	2.64
746.567	12.85	PC(15:0/18:1(11Z))	$C_{41}H_{80}NO_8P$	1.71	0.008	1.66	0.73
306.264	6.09	Ustilic acid A	$C_{16}H_{32}O_4$	1.63	0.000	67.19	6.07
796.581	13.73	PS(16:0/18:1(9Z))	$C_{40}H_{79}N_2O_{10}P$	1.55	0.032	4.97	2.31
290.269	7.40	16-Hydroxy hexadecanoic acid	$C_{16}H_{32}O_3$	1.43	0.000	8.54	3.09
433.019	0.78	Rhamnazin 3-O-sulfate	$C_{17}H_{14}O_{10}S$	1.42	0.003	3.46	1.79
410.267	14.24	Tetradecanoylcarnitine	$C_{21}H_{41}NO_4$	1.41	0.017	3.68	1.88
318.300	8.55	18-hydroxy stearic acid	$C_{18}H_{36}O_3$	1.41	0.038	0.77	-0.38
408.251	14.30	PC(11:0/0:0)[U]	$C_{19}H_{40}NO_7P$	1.33	0.019	3.59	1.84
747.591	14.26	DG(22:0/22:6(4Z,7Z,10Z,13Z,16Z,19Z)/0:0)	$C_{47}H_{80}O_5$	1.06	0.037	0.75	-0.42
192.159	1.13	Nonanoic acid, 8-hydroxy-, (R)-; (-)-(R)-8-Hydroxynonanoic acid	$C_9H_{18}O_3$	1.01	0.000	569.95	9.15
352.321	12.61	13,16,19-docosatrienoic acid	$C_{22}H_{38}O_2$	1.01	0.043	0.84	-0.24

Supplemental Table S6. Differential Organic Heterocyclic Compounds metabolites.

m/z	Retention time (min)	Metabolites	Formula	VIP	P-value	FC	log2(FC)
729.230	0.85	Citbismine C	$C_{37}H_{36}N_2O_{11}$	9.11	0.007	1.65	0.73
181.090	2.01	SK&F 91581	$C_8H_{14}N_4S$	3.81	0.021	2.66	1.41
370.083	0.90	Lansoprazole	$C_{16}H_{14}F_3N_3O_2S$	2.75	0.004	1.66	0.73
259.081	0.85	Ranunculin	$C_{11}H_{16}O_8$	2.66	0.009	1.40	0.49
281.071	0.91	SULFAMETER	$C_{11}H_{12}N_4O_3S$	1.75	0.002	4.90	2.29
289.057	0.91	Furamizole	$C_{12}H_8N_4O_5$	1.47	0.003	3.07	1.62
513.103	5.08	Cefpirome	$C_{22}H_{22}N_6O_5S_2$	1.05	0.000	0.17	-2.59
431.005	0.95	5-(3-Buten-1-ynyl)-2,2'-bithiophene	$C_{12}H_8S_2$	1.03	0.002	0.57	-0.81

Supplemental Table S7. Other differential metabolites.

m/z	Retention time (min)	Metabolites	Formula	VIP	P-value	FC	log2(FC)
324.217	2.09	Latanoprost Lactol	$C_{18}H_{26}O_4$	5.25	0.0134	1.28	0.35
281.070	0.78	SULFAMONOMETHOXINE	$C_{11}H_{12}N_4O_3S$	2.76	0.001	6.81	2.77
439.076	0.95	5'-Butyrylphosphouridine	$C_{13}H_{19}N_2O_{10}P$	2.39	0.018	1.34	0.42
443.123	0.79	S-(4-Nitrobenzyl)glutathione	$C_{17}H_{22}N_4O_8S$	2.32	0.000	6.42	2.68
405.028	1.13	{2-hydroxy-5-[3-(4-methoxy-1-benzofuran-5-yl)-3-oxopropanoyl]phenyl}oxidanesulfonic acid	$C_{18}H_{14}O_9S$	1.49	0.000	46044312.16	25.46
520.073	4.79	4-(Cytidine 5'-diphospho)-2-C-methyl-D-erythritol	$C_{14}H_{25}N_3O_{14}P_2$	1.37	0.007	2.61	1.38
426.009	1.14	CDP	$C_9H_{15}N_3O_{11}P_2$	1.20	0.001	30206917.73	24.85
406.352	9.01	Aminopentol	$C_{22}H_{47}NO_5$	1.00	0.033	0.75	-0.41

Supplementary Figure S1. Heatmap analysis of microbial community composition at the class and family levels. (A) Heatmap of microbial community composition at the class level. (B) Heatmap of microbial community composition at the family level. Colors indicate relative abundance.

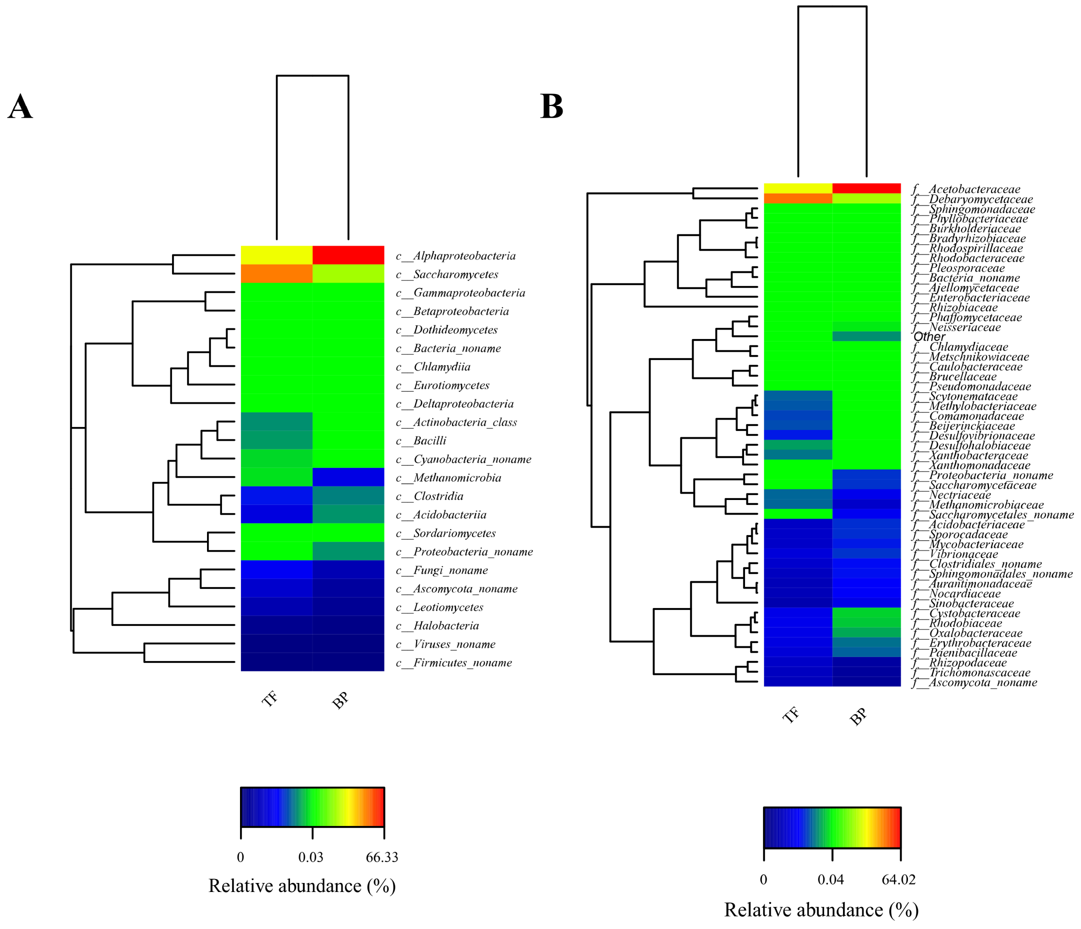

Supplementary Figure S2. GO functional classification of metagenomic genes in the BP and TF systems.

(A) GO functional classification of annotated genes in the black tea–sucrose system, BP. (B) GO functional classification of annotated genes in the theaflavin–sucrose system, TF. GO terms were assigned to three major categories: biological process, cellular component, and molecular function. Bar heights indicate the proportion or number of genes assigned to each GO term.

A

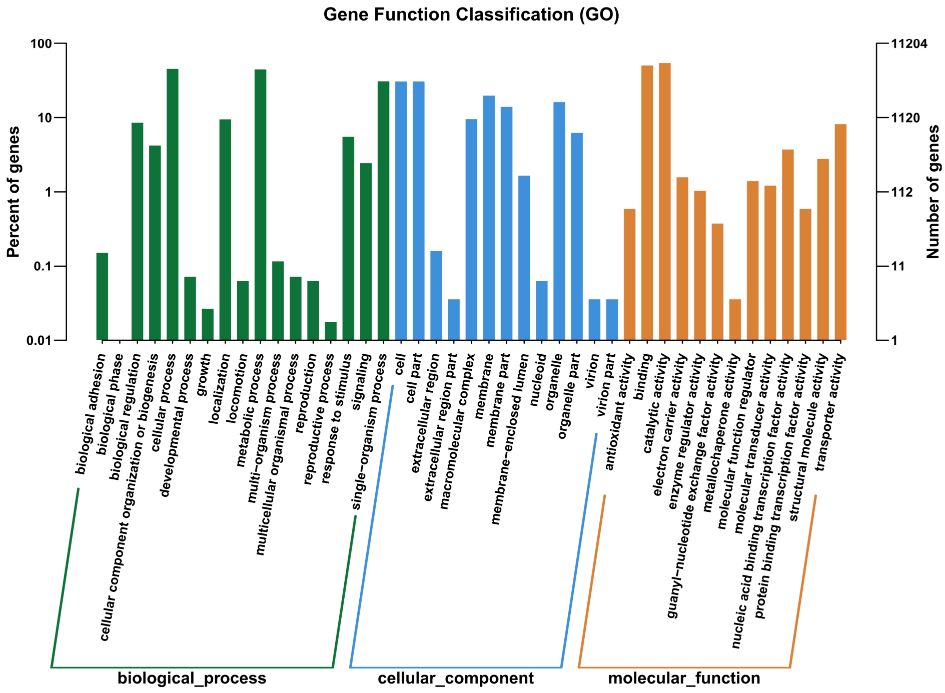

B

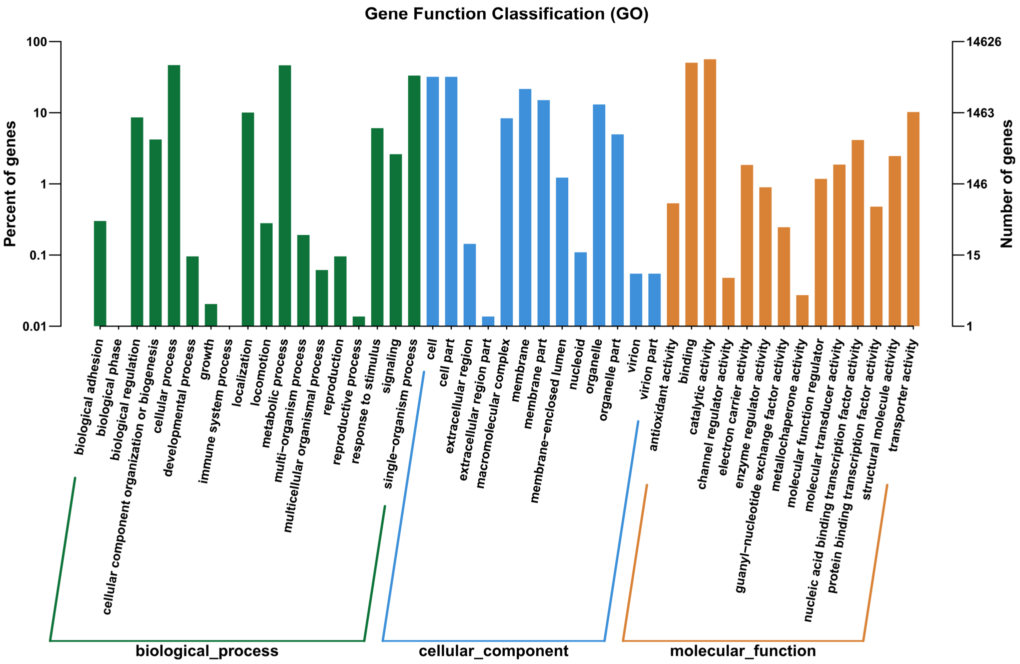

Supplementary Figure S3. The photos of novel isolated Meyerozyma sp. C3 yeast.

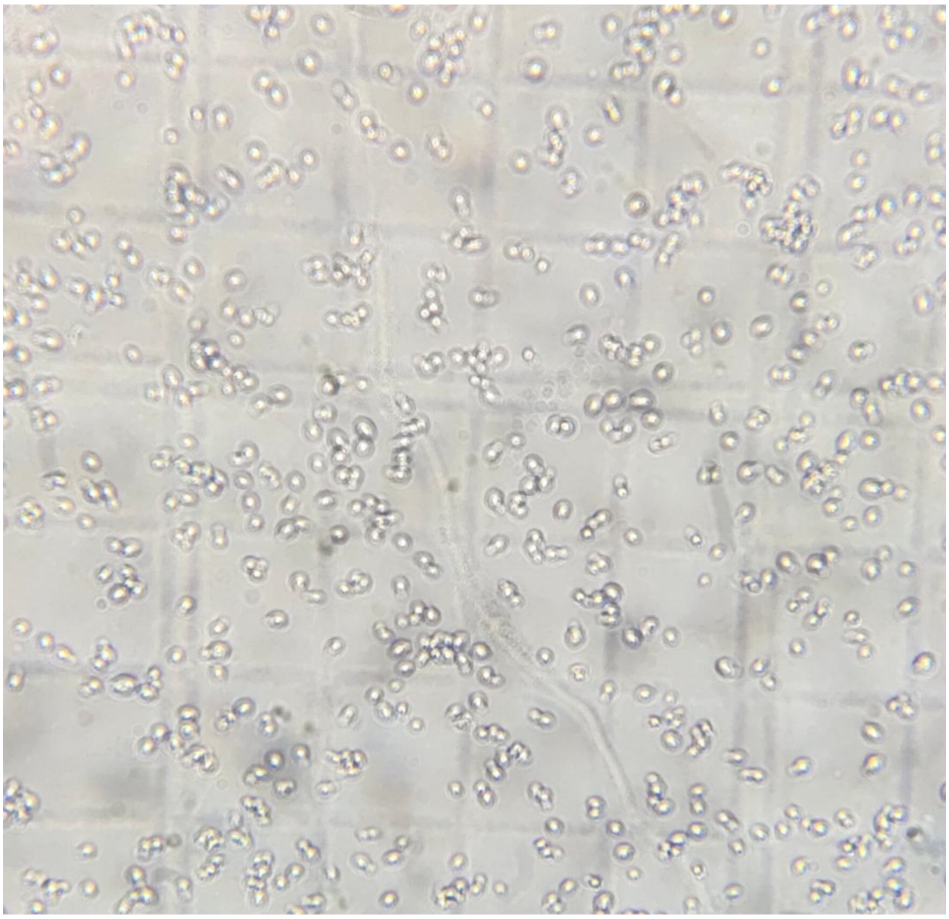

Supplementary Figure S4. Yeast separation process.

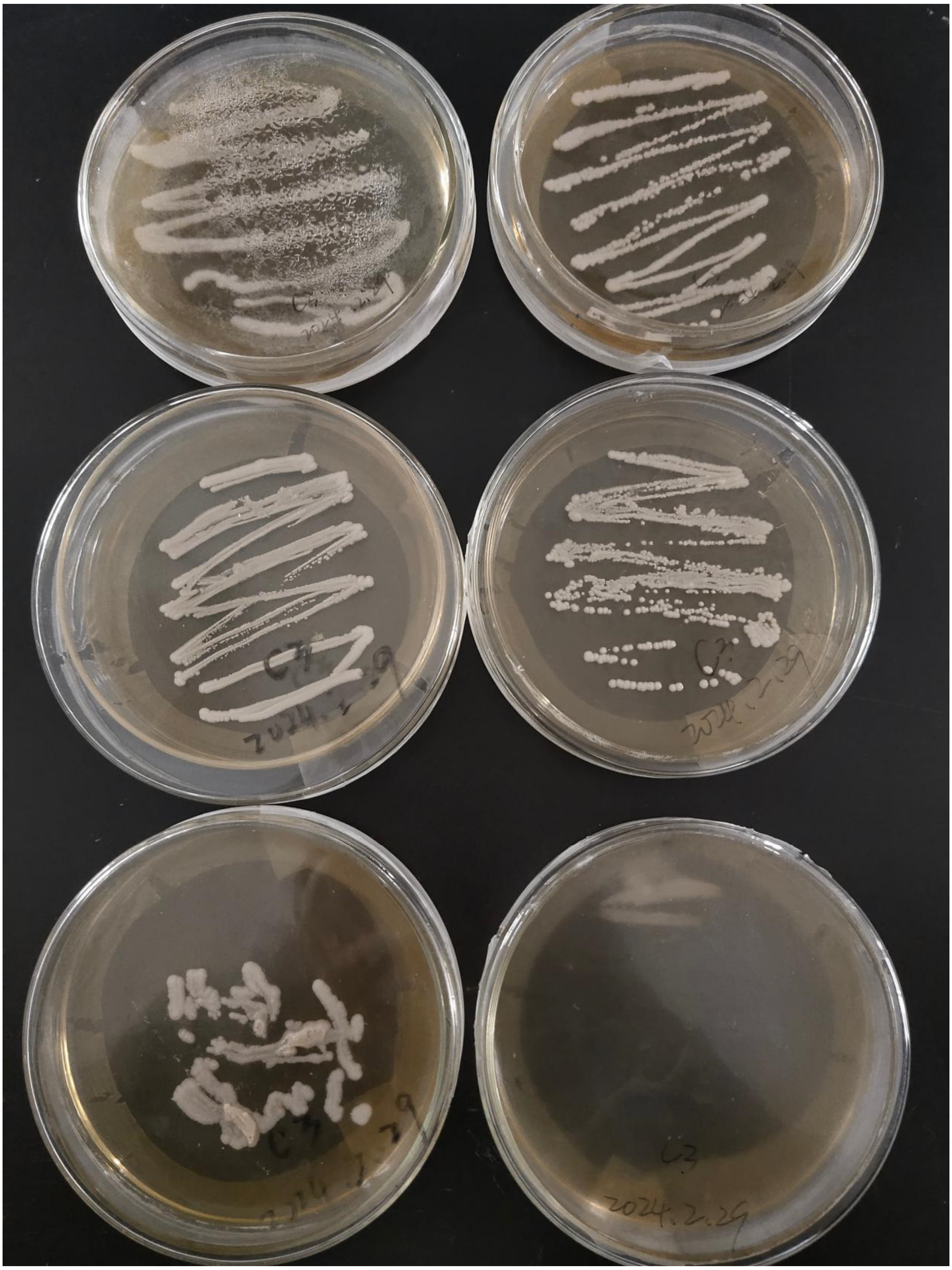

Supplement: Supplementary file 1 [file foods-15-03453-s001.zip › foods-4549845-supplementary.pdf]
